# Berberine Inhibits Telomerase Activity and Induces Cell Cycle Arrest and Telomere Erosion in Colorectal Cancer Cell Line, HCT 116

**DOI:** 10.3390/molecules26020376

**Published:** 2021-01-13

**Authors:** Muhammad Azizan Samad, Mohd Zuwairi Saiman, Nazia Abdul Majid, Saiful Anuar Karsani, Jamilah Syafawati Yaacob

**Affiliations:** 1 Institute of Biological Sciences, Faculty of Science, Universiti Malaya, Kuala Lumpur 50603, Malaysia; azizan_12@siswa.um.edu.my (M.A.S.); zuwairi@um.edu.my (M.Z.S.); saiful72@um.edu.my (S.A.K.); 2 Centre for Research in Biotechnology for Agriculture (CEBAR), Faculty of Science, Universiti Malaya, Kuala Lumpur 50603, Malaysia

**Keywords:** colorectal cancer, HCT 116, telomerase, telomerase inhibitor, berberine, downregulation, cell cycle arrest, telomere erosion

## Abstract

Colorectal cancer (CRC) is the most common cancer among males and females, which is associated with the increment of telomerase level and activity. Some plant-derived compounds are telomerase inhibitors that have the potential to decrease telomerase activity and/or level in various cancer cell lines. Unfortunately, a deeper understanding of the effects of telomerase inhibitor compound(s) on CRC cells is still lacking. Therefore, in this study, the aspects of telomerase inhibitors on a CRC cell line (HCT 116) were investigated. Screening on HCT 116 at 48 h showed that berberine (10.30 ± 0.89 µg/mL) is the most effective (lowest IC_50_ value) telomerase inhibitor compared to boldine (37.87 ± 3.12 µg/mL) and silymarin (>200 µg/mL). Further analyses exhibited that berberine treatment caused G_0_/G_1_ phase arrest at 48 h due to high cyclin D1 (CCND1) and low cyclin-dependent kinase 4 (CDK4) protein and mRNA levels, simultaneous downregulation of human telomerase reverse transcriptase (*TERT*) mRNA and human telomerase RNA component (*TERC*) levels, as well as a decrease in the TERT protein level and telomerase activity. The effect of berberine treatment on the cell cycle was time dependent as it resulted in a delayed cell cycle and doubling time by 2.18-fold. Telomerase activity and level was significantly decreased, and telomere erosion followed suit. In summary, our findings suggested that berberine could decrease telomerase activity and level of HCT 116, which in turn inhibits the proliferative ability of the cells.

## 1. Introduction

Colorectal cancer (CRC) (10.2% incidence) is the third most common cancer among males and females worldwide after lung (11.6% incidence) and breast cancers (11.6% incidence) [1]. CRC is often associated with an elevated telomerase level [2] and activity [3]. Telomerase is an enzyme responsible for adding the repetitive sequence (TTAGGG) at telomeric ends for telomere maintenance in humans [4]. Telomerase is a prognostic marker of CRC progression [3,5]. Patients with high telomerase activity have poorer prognosis and disease-free survival rates compared to those with low and moderate telomerase activity.

It has been previously suggested that human telomerase consists of six subunits, namely the human telomerase reverse transcriptase (hTERT or TERT), human telomerase RNA component (*hTR* or *TERC*), telomerase-associated protein 1 (TEP1), heat shock protein 90 (HSP90), prostaglandin E synthase 3 (P23), and dyskerin (DKC) [6]. However, recent review articles discussed that telomerase, which is synthesized during the S phase, has more than just six subunits [7]. It has been shown that telomerase activity changed proportionally with the level of TERT but not with the other five subunits [6]. This could be due to the fact that TERT is the catalytic subunit of telomerase [8]. *TERC* is a functional RNA sequence that is responsible for providing a template for 3′TTAGGG5′ addition at telomere ends [4]. It was also stated that TERT and *TERC* are enough for telomerase to function [6]. Due to this reason, TERT and *TERC* serves as the prime target for telomerase level and activity inhibition. The most recent publication on the telomerase structure of the colorectal cancer cell line, HCT 116, surprisingly revealed that there were no TEP1, P23, and HSP90 subunits present [9]. This evidence agrees with the previous statement, whereby only TERT and *TERC* are required for telomerase to function. It might also suggest that telomerase could adopt different structures.

Since telomerase is the hallmark of CRC, targeting telomerase could be a potential treatment for telomerase-positive cancers, specifically CRC in this case. Many natural products have been listed as telomerase-targeting compounds or, in other words, telomerase inhibitors [10]. Boldine, silymarin, and berberine are some that are included in the list. Boldine is an alkaloid that could be found abundantly in the leaves of *Peumus boldus*, which has been used as a traditional medicine in Chile [11]. Silymarin is a collective name of seven flavonolignans, namely silydianin, isosilchristin, silychristin, silibinin (or silybin) A, isosilibinin (or isosilybin) A, silibinin B, and isosilibinin B, as well as one flavonoid named taxifolin [12,13], which is present in *Silybum marianum* (milk thistle) [14]. On the other hand, berberine is an alkaloid that can be found in the outer bark, roots, and rhizhome of *Berberis* spp, such as *B. aristata*, *B. aquafolium*, and *B. vulgaris* [15]. Besides that, berberine could also be extracted from *Tinospora* spp, such as *T. sinensis* and *T. cordifolia* [16]. These compounds have been reported to be able to downregulate telomerase activity and/or level in various cancer cell lines [10].

Telomerase is highly expressed in proliferative cells, such as cancer, stem, and gamete cells, but not in normal cells [17]. However, telomerase activity is higher in cancer cells compared to most stem cells except for embryonic stem cells [18]. It is challenging to determine whether telomerase inhibition will affect gamete and stem cells as cancer cells are affected; however, due to the shorter length of telomere as well as the difference of the metabolism in cancer cells, it would have undergone apoptosis even before it could adversely affect germ line and stem cells [17]. The aim of this study was to investigate the effects of telomerase inhibitor compound treatment on the cell cycle, doubling time (t_d_), telomerase activity, and level as well as on the telomere length of a colorectal cancer cell line, using HCT 116 as a model.

## 2. Results

### 2.1. Effect of Different Time Points on the Percentage of Cell Cycle Distribution and Relative Telomerase Activity (RTA)

**Figure 1:**
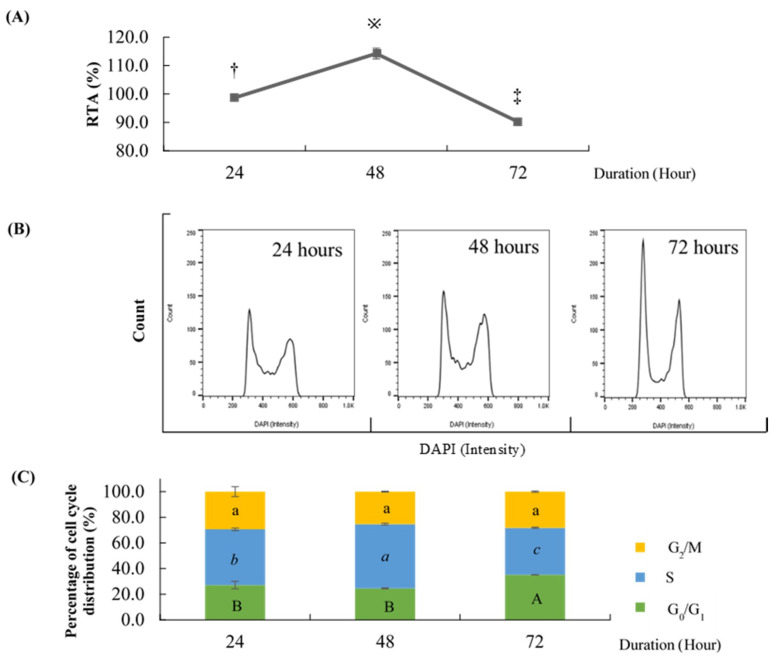
Effects of different time points on relative telomerase activity (RTA) and percentage of HCT 116 cell cycle distribution. (**A**) Telomerase activity of HCT 116 harvested at different time points relative to telomerase-positive control (HEK293). Values are mean ± standard error of triplicate. Mean with different symbols (†, ※, or ‡) differ significantly at *p* < 0.001. (**B**) Representative cell cycle histogram of HCT 116 harvested at different time points stained with DAPI and analyzed by flow cytometry. (**C**) Percentage of cell cycle distribution of HCT 116 at different time points analyzed by FlowJo version 10. Values are mean ± standard error of triplicate. Mean with different letters differ significantly at *p* < 0.05 (subject to different cell cycle phases). G: Growth, S: Synthesis, M: Mitosis.

It has been hypothesized that telomerase activity peaked during S phase [19,20], thus it was necessary to determine the time point that possessed the highest percentage of S phase. Cell cycle analysis was done on HCT 116 cells at different time points to determine the percentage of cell cycle phases (Figure 1). Here, 72 h exhibited the highest percentage of G_0_/G_1_ phase (35.13 ± 0.25%) compared to 24 h and 48 h (27.17 ± 5.14% and 24.63 ± 0.67%, respectively). Based on the results, the percentage of S phase was significantly highest at 48 h (50.10 ± 1.15%), followed by 24 h (43.43 ± 1.59%) and 72 h (36.70 ± 0.75%). The RTA of HCT 116 indeed peaked at 48 h (114.26 ± 1.26%) followed by 24 h (98.69 ± 0.42%) and 72 h (90.24 ± 0.91%). All three time points gave an insignificant percentage of G_2_/M phase. Thus, 48 h was chosen for the treatment duration for subsequent experiments since it showed the highest percentage of S phase and RTA.

### 2.2. Effect of Different Telomerase Inhibitor Compounds on IC_50_

HCT 116 cultured in 96-well plates treated with different concentrations of boldine, silymarin, or berberine for 48 h were subjected to SRB assay to determine the percentage of inhibition and the results were interpreted as IC_50_ (compound concentration required to inhibit HCT 116 by 50%). Berberine was found to be the most effective, as it showed the lowest IC_50_ compared to boldine and silymarin (Table 1). Hence, berberine was chosen to be used for subsequent analysis.

### 2.3. Effect of Berberine Treatment on Cell Cycle Distribution

**Figure 2:**
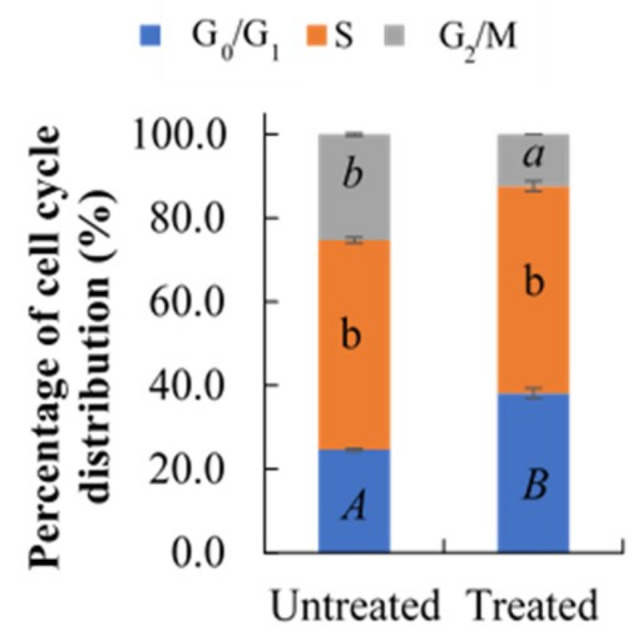
Effect of 10.54 µg/mL berberine treated HCT 116 at 48 h on the percentage of cell cycle distribution. Values are mean ± standard error of triplicate. Mean with different letters differ significantly at *p* < 0.001 (subject to different cell cycle phases).

The cells were treated with berberine for 48 h and were analyzed for cell cycle distribution. Treatment with berberine was observed to cause a cell cycle arrest at G_0_/G_1_ phase, as shown by the significant increase in the number of cells in G_0_/G_1_ compared to that of untreated sample (Figure 2). The cells in S phase remained unchanged while the cells also did not proceed to G_2_/M, as indicated by a significant decrement of G_2_/M.

**Figure 3:**
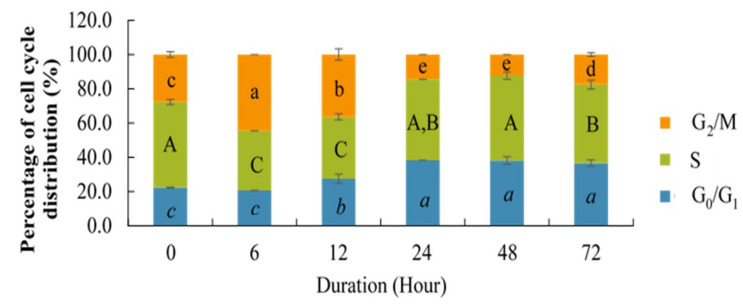
Changes in the percentage of HCT 116 cells in G_0_/G_1_, S, and G_2_/M phases at different durations following treatment with 10.54 µg/mL berberine. Values are mean ± standard error of triplicate. Mean with different letters differ significantly at *p* < 0.001 (subject to different cell cycle phases).

The cells were also exposed to berberine at various time points, to determine if the effects observed were time dependent. Based on Figure 3, the G_0_/G_1_ percentage started to increase gradually after 12 h and became constant after 48 h. The percentage of cells in S phase significantly decreased from 0 to 6 h after treatment, then started to increase again from 12 to 24 h, then became stable at 48 h followed by a slight decrement at 72 h; however, the percentages of S phase at 24 and 72 h were not significantly different. G_2_/M started to increase from 0 to 6 h after treatment, then decreased from 12 to 24 h, stabilized at 48 h, and then slightly increased at 72 h. This scenario showed that the effect of berberine on cell cycle was time dependent.

### 2.4. Effect of Berberine Treatment on Cell Growth and Berberine Cellular Localization

**Figure 4:**
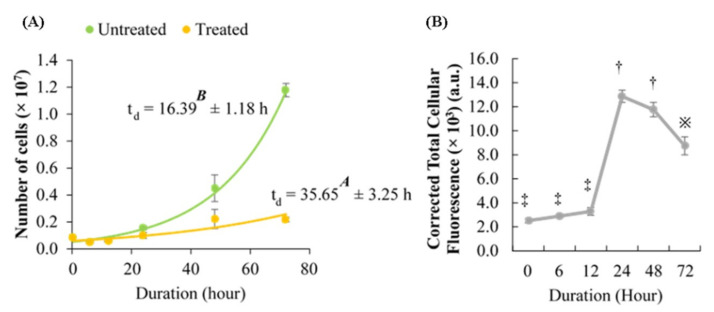
(**A**) Growth curve of untreated and 10.54 µg/mL berberine-treated HCT 116. Values are mean ± standard error of biological quadruplicate. t_d_: doubling time. (**B**) Corrected Total Cellular Fluorescence (CTCF) of berberine-treated HCT 116 at different durations. Values are mean ± standard error of biological triplicate. a.u.—arbitrary unit. Mean with different letters or symbols (‡, †, or ※) differ significantly at *p* < 0.001.

The time-dependent effect of berberine on HCT 116 could also be observed microscopically. Based on Table 2, it was observed that the cell growth was inhibited by berberine treatment. The difference in growth could be clearly observed at 48 and 72 h. Figure 4A also showed that berberine treatment caused the doubling time (t_d_) of HCT 116 to be delayed. The green fluorescence intensity observed under the UV channel peaked at 24 h, then decreased at 72 h (see Figure 4B). It was also noted that at 24 h, the nuclei appeared as black round hollows surrounded by green fluorescence, which potentially indicates that berberine had permeated and accumulated in the cytoplasm. The black hollows were no longer observed after 48 and 72 h, suggesting that berberine had permeated and accumulated in the nuclei. Untreated HCT 116 did not fluoresce at all under the UV channel and the image only appeared as black.

### 2.5. Effect of Berberine Treatment on Relative Telomerase Activity (RTA)

**Figure 5:**
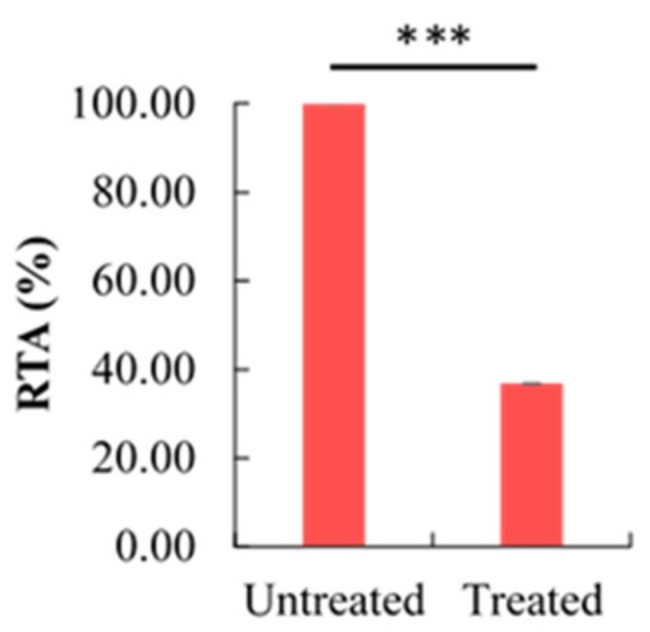
Effect of 10.54 µg/mL berberine-treated HCT 116 at 48 h on relative telomerase activity (RTA). Values are mean ± standard error of triplicate. Means with *** (triple asterisks) differ significantly at *p* < 0.001.

**Figure A1:**
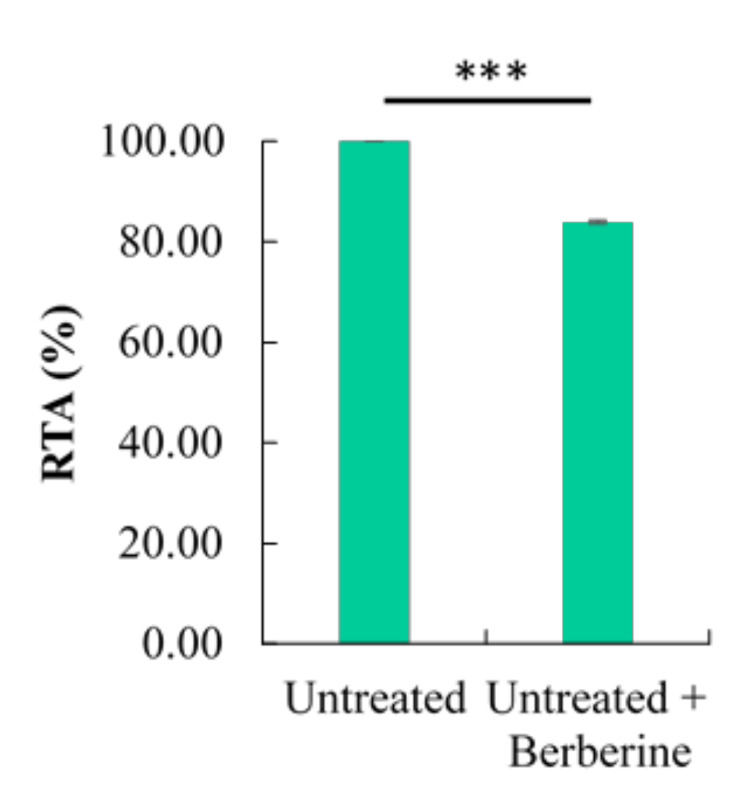
Cell-free telomerase inhibition by direct addition of berberine in the TRAP reaction mixture of untreated HCT 116 cell lysate. The relative telomerase activity (RTA) of untreated HCT 116 cell lysate added with berberine was 83.90 ± 0.34%. Berberine only decreased the RTA of untreated HCT 116 cell lysate by 16.10%. Values are mean ± standard error of triplicate. Mean with *** (triple asterisks) differ significantly at *p* < 0.001.

Berberine-treated HCT 116 at 48 h were analyzed by using *TELOTAGGG* Telomerase PCR ELISA. The telomerase activity was observed to significantly decrease with berberine treatment (Figure 5). Compared to untreated HCT 116, the RTA in berberine-treated HCT 116 was decreased by 63.22%. To evaluate cell-free telomerase inhibition, berberine was added into the TRAP reaction mixture to the same final concentration and the results showed that berberine only decreased the RTA by 16.10% (see Figure A1). This might suggest that the inhibition of telomerase–TTAGGG interaction was not the only reason for the decrement in the RTA, whereby the level of telomerase might be affected, which caused the RTA to decrease even further. As such, we are interested in investigating the effect of berberine on TERT and *TERC* levels.

### 2.6. Effect of Berberine Treatment on CCDN1, CDK4, TERT, and TERC RNA Levels

**Figure 6:**
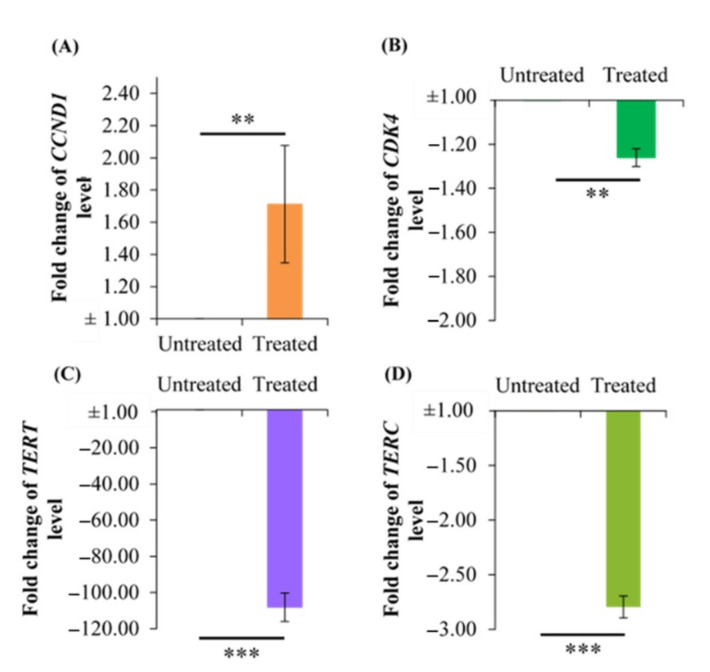
Effect of 10.54 µg/mL berberine-treated HCT 116 at 48 h on (**A**) *CCND1*, (**B**) *CDK4*, (**C**) *TERT*, and (**D**) *TERC* levels. Changes in RNA levels are expressed as fold change in RNA levels relative to untreated HCT 116 at 48 h. Values are mean ± standard error of quadruplicate. Mean with ** (double asterisks) or *** (triple asterisks) differ significantly at *p* < 0.01 or *p* < 0.001, respectively.

In cell cycle progression, CCND1 and CDK4 are important regulators for G_1_ to S phase transition, whereby the binding of CCND1 to CDK4 is a rate-limiting event [21]. Since G_0_/G_1_ phase arrest was observed (Figure 2), we are keen to know the effect of berberine on the RNA levels of these regulators. Based on the RT-PCR results (Figure 6), surprisingly, 10.54 µg/mL berberine treatment caused *CCND1* level to be upregulated by 1.71-fold. Besides that, *CDK4*, *TERT*, and *TERC* levels were downregulated by 1.26-, 108.15-, and 2.80-fold, respectively.

### 2.7. Effect of Berberine Treatment on CCND1, CDK4, and TERT Protein Levels

**Figure 7:**
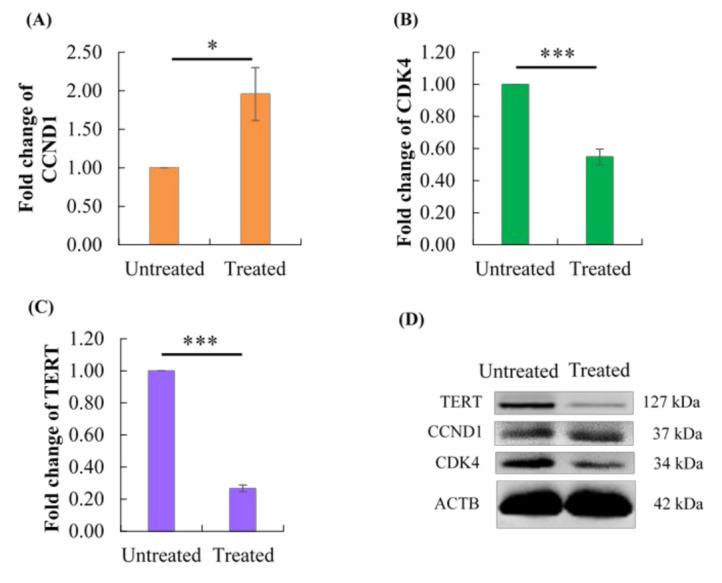
Effect of 10.54 µg/mL berberine-treated HCT 116 at 48 h on (**A**) CCND1, (**B**) CDK4, and (**C**) TERT protein levels. Changes in CCND1, CDK4, and TERT protein levels are expressed as fold change in CCND1, CDK4, and TERT signal intensities, respectively, normalized to ACTB relative to untreated HCT 116 at 48 h. (**D**) Representative Western blot images of TERT, CCND1, CDK4, and ACTB. Values are mean ± standard error of triplicate. Mean with * (single asterisk) or *** (triple asterisks) differ significantly at *p* < 0.05 or *p* < 0.001, respectively.

Parallel to the downregulation of *CDK4* and *TERT* mRNA levels, validation by Western blot results (Figure 7) also showed that berberine treatment at 48 h caused CDK4 and TERT protein levels to decrease significantly by 45.16% and 73.29%, respectively. Besides this, the CCND1 protein level increased significantly by 95.77%.

### 2.8. Effect of Berberine Treatment on Relative Telomere Length

**Figure 8:**
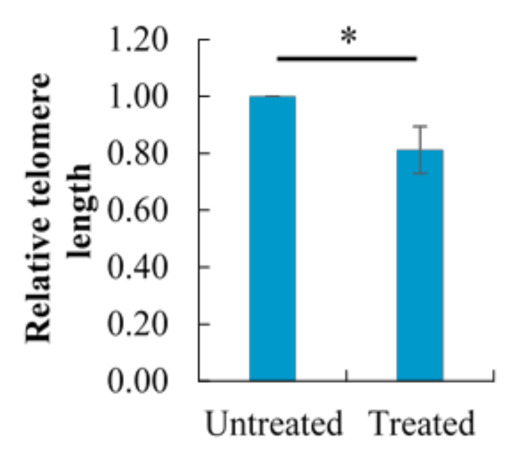
Effect of 10.54 µg/mL berberine-treated HCT 116 at 48 h on the relative telomere length. Changes in telomere length are expressed as fold change relative to untreated HCT 116 at 48 h. Values are mean ± standard error of octuplicate. Mean with * (single asterisk) differ significantly at *p* < 0.05.

Consistent with the significantly impaired telomerase activity and level by berberine treatment, telomere attrition also occurred as anticipated (Figure 8), whereby the relative telomere length of berberine-treated HCT 116 after 48 h was 0.81 ± 0.08, compared to untreated HCT 116 (with a relative telomere length of 1.0).

## 3. Discussion

The cytotoxicity of drugs could be classified into cytotoxic (less than 2 µg/mL), moderately cytotoxic (between 2 to 89 µg/mL), and not cytotoxic (more or equal to 90 µg/mL) based on their IC_50_ values [22]. In the present study, berberine and boldine were found to be moderately cytotoxic, while silymarin was not cytotoxic to HCT 116 after 48 h of treatment. Since silymarin did not reach IC_50_, the value was reported as >200 µg/mL, which was greater than the highest tested concentration as suggested by the National Cancer Institute [23].

In several telomerase-positive cancer cell lines, berberine treatment has been shown to cause G_0_/G_1_ phase arrest in MCF-7 (breast cancer) after 48 h [24], Huh-7 and HEPG2 (liver cancers) in a dose-dependent manner after 24 h [25], and A2780 (ovarian cancer) after 48 h of treatment [26]. Either too high or too low CCND1 levels would compromise cell cycle progression [27]. In the present study, it was revealed that an abnormal level of cell cycle regulators, in this case high CCND1 levels and low CDK4 levels, would lead to G_0_/G_1_ phase arrest in HCT 116. Since the binding of CCND1 to CDK4 is a rate-limiting event in G_1_ to S phase transition [21], theoretically, the effect of berberine on G_0_/G_1_ arrest would depend mainly on these two proteins instead of other cyclins and CDKs. Besides that, telomerase is mostly active during S phase, hence the reason the study was focused more on CCND1 and CDK4 instead of other cyclins and CDKs. Due to this reason, the experiments were designed in such flow, so that the study was more focused to the factors that are associated within the telomerase aspect.

Wang, et al. [28] reported that G_0_/G_1_ arrest in U87 and LN229 (brain cancer) was caused by the downregulation of *miR-21*, which caused a decrement of STAT3 phosphorylation and protein level, thus causing the TERT protein level to decrease. In a separate study, berberine showed the ability to repress the *miR-21* level in oral squamous cell carcinoma cancer stem cell lines (OECM-1 and SAS), which decreased aldehyde dehydrogenase 1 (ALDH1) activity, cell migration, invasion capability, and self-renewal [29]. Moreover, G_0_/G_1_ phase arrest in HL-60 (leukemia) is also associated with a decrement of telomerase activity as well as *TERT* and *TERC* levels [30]. It was also previously shown that berberine treatment for 24 h induced G_0_/G_1_ phase arrest in HCT 116 in a dose-dependent manner [31]. Based on these previous findings, it would be fair to remark that the inhibition of cell proliferation (Figure 4) and G_0_/G_1_ phase arrest in HCT 116 induced by berberine treatment were not exclusively caused by the downregulation of TERT.

Based on various references, the average duration of untreated HCT 116 cell cycle was about 17 h (16.88 ± 2.56 h) [32,33,34,35,36]. Therefore, theoretically, the second round of HCT 116 cell cycle would take place approximately after 34 h. Based on Figure 4A, the average doubling time (t_d_) for untreated HCT 116 was 16.39 ± 2.36 h, similar to the average t_d_ of previous findings. The average t_d_ of berberine-treated HCT 116 was 35.65 ± 6.50 h, significantly higher than the average t_d_ of untreated HCT 116. Data analysis revealed that the t_d_ of berberine-treated HCT 116 was overdue by approximately two-fold (2.18-fold).

The localization of berberine in nuclei after 48 h further strengthens our justification of choosing this time point for further investigation. This is due to the fact that the transcription, synthesis, and activity of telomerase mainly takes place in the nucleus [7,37]. Since we are targeting the telomerase, berberine should be present in the nucleus for telomerase inhibition to occur. Here, we have demonstrated that berberine cellular localization is time dependent. Similarly, berberine cellular localization has also been shown to be concentration [38] and cell line dependent [39].

It was previously established that berberine treatment on HCT 116 inhibits cell proliferation in a dose- and time-dependent manner [31,40], and induced apoptosis in a dose-dependent manner at 24 [40] and 72 h [31]. Downregulation of TERT levels has been associated with a decrement in telomerase activity [19,30,41] and vice versa [42]. Berberine intervenes in the function of telomerase through binding to the telomeric G-quadruplex structure [43,44], thus preventing the interaction between telomerase and telomere [45]. It was revealed in previous studies that berberine had the potential to regulate gene transcription [46,47]. Other than that, the decrement of the TERT protein level was often associated with downregulation of *TERT* mRNA [28] and vice versa [42]. Berberine could downregulate the TERT protein level in the non-small-cell lung cancer cell line, A549 [48], and cervical cancer cell lines, SiHa and HeLa [15]. Previous studies mostly focused only on the potential of berberine in downregulation of the TERT level; however, in the present study, we demonstrated that berberine could also downregulate the *TERC* level.

Berberine has been shown to decrease the telomerase level and activity, which is significantly followed by telomere erosion. Interestingly, by conducting telomere restriction fragment (TRF) analysis, Xiong et al. [49] revealed that prolonged exposure to berberine for 16 days did not significantly cause telomere shortening in SiHa and HL-60. This could possibly be due to the limitation of TRF, which could potentially overestimate the telomeric length compared to RT-PCR, which is more sensitive and specific [50]. Other than the potential to regulate gene transcription, berberine could interact directly with the telomeric G-quadruplex, whereby the interaction occurred between the negatively charged oxygen of guanine and positively charged nitrogen of berberine [51] with a molar ratio of 2:1 (berberine to G-tetrad) [52], as it stabilized the structure by increasing the melting temperature and as a result, telomerase activity was inhibited [53]. Berberine has been extensively studied in various cancer cell lines; however, in animal models, evidence to support the effects of berberine as a telomerase inhibitor on colorectal cancer is still lacking [54]. Berberine is effective at inhibiting polyps formation [55] and colon tumorigenesis in mice [40], however, currently, there is no in vivo study that validates berberine’s presence in the nucleus of in vivo cancer cells of mice after oral administration of berberine. Since berberine is currently used in clinical trials (NCT03281096 and NCT03333265), there is an urge to elucidate berberine mechanisms as an anti-colorectal cancer drug.

In this study, cells from four flasks (biological replicates) were pooled to obtain sufficient material for several different analyses. All extractions and preparations were also performed from the same set of samples for the sake of uniformity. Although pooling of samples may rightfully raise some concerns, it is still widely viewed as a valid method, saves cost, and reduces biological variance, while not seriously affecting the results when compared to un-pooled samples [56,57,58]. Biological replicates and independent experiments are important to validate the effect of any anticancer drug due to the existence of heterogeneity the within cancer cell population. It was fully acknowledged that the lack of an independent experiment is a limitation in the current study. Though the potential mechanism of telomerase inhibition by berberine using HCT 116 as a model has been shown in the current study, further deeper understanding is vital to elucidate the potential of berberine as a telomerase inhibitor in CRC.

## 4. Materials and Methods

### 4.1. Reagents, Primers, and Antibodies

Berberine was diluted in autoclaved Milli-Q^®^ water and sterilized by filtration through a Minisart^®^ 0.22 µm polyethersulfone filter syringe (Sartorius Stedim Biotech, Göttingen, Germany). Boldine and silymarin was dissolved in dimethylsulfoxide (DMSO) and filtered as well. All primers were synthesized by Integrated DNA Technologies (IDT, Coralville, IA, USA). Monoclonal antibodies of CCND1 (A-12), CDK4 (DCS-31), TERT (A-6), and β-actin (ACTB) (AC-15) as well as mouse IgG kappa binding protein conjugated to horseradish peroxidase (m-IgGκ BP-HRP) were obtained from Santa Cruz Biotechnology (Santa Cruz, Dallas, TX, USA). Molecular-grade absolute ethanol (Merck, Darmstadt, Germany) was diluted with autoclaved Milli-Q^®^ water. All reagents were from Sigma-Aldrich unless mentioned otherwise.

### 4.2. Cell Culture and Harvesting

Colorectal cancer cell line, HCT 116, was cultured in Roswell Memorial Institute (RPMI) medium 1640 (Nacalai Tesque, Kyoto, Japan) supplemented with 10% fetal bovine serum (Tico Europe Ltd., Amstelveen, Netherlands), 1% Antibiotic-Antimycotic solution (Nacalai Tesque, Kyoto, Japan), maintained at 37 °C in a humidified 5% CO_2_-enriched atmosphere (ESCO, Horsham, PA, USA). Cells were rinsed by using 1× phosphate-buffered saline (PBS) (Nacalai Tesque, Kyoto, Japan), repeated three times, and cells were detached by using trypsin-EDTA (Life Technologies, Burlington, Canada), followed by RPMI addition to stop the trypsinization; cells were pelleted by centrifugation at 1000 rpm, resuspended in RPMI for sub-culturing or seeding purpose, or 1× PBS was used to resuspend for harvesting as a washing step, followed by second-time pelleting of the cells. During harvesting, after observation under the microscope and cell count was conducted, cells from four flasks were pooled and pelleted in the amount as required for further analyses. The microcentrifuge tubes containing a pelleted known number of cells were immediately flash frozen in liquid nitrogen before storage in −80 °C until further usage.

### 4.3. Cell Cycle Analysis

In total, 1 × 10^6^ pelleted cells in 2 mL microcentrifuge tube were washed with 1 mL of sample buffer (1× PBS containing 1 g/L glucose) once by vortexing briefly, followed by centrifugation at 17,000× *g* for 5 min. The supernatant was removed and about 0.1 mL was left in the tube. Then, 1 mL of 70% ethanol (−20 °C) was slowly added, 100 µL at a time, drop by drop while vortexing. The suspension was stored at 4 °C for 24 h. After fixation, 1 mL of sample buffer was added while vortexing, followed by centrifugation at 17,000× *g* for 10 min. Staining solution made up of 0.5 µg/mL 4′,6 -diamidino-2-phenylindole (DAPI) (Miltenyi Biotec, Bergisch Gladbach, Germany), 5 µg/mL RNase A, and 0.1% Tween 20 (Classic Chemicals, Shah Alam, Selangor, Malaysia) in 1× PBS was prepared immediately before incubation. The supernatant was removed, and 1 mL of staining solution was added followed by vortexing and incubation at room temperature for 40 min. Flow cytometry was done by using a MACSQuant^®^ Analyzer (Miltenyi Biotec, Bergisch Gladbach, Germany) following the protocol suggested by Miltenyi Biotec with optimizations. The fluorescence channel was set to V1 and measured by linear acquisition. The V1 voltage was set to 334 V and the trigger was set to 128.00. The number of events was set to 10,000 events for each replicate. The data was analyzed by using FlowJo version 10.

### 4.4. Sulforhodamine B (SRB) Assay

In total, 5000 cells/100 µL per well were seeded in 96-well plates, incubated at 37 °C in a humidified 5% CO_2_-enriched incubator for 24 h before compound treatments. The spent medium was pipetted out and replaced with 150 µL of RPMI containing either boldine, silymarin, or berberine at various concentrations (0.20, 0.39, 0.78, 1.56, 3.13, 6.25, 12.50, 25.00, 50.00, 100.00, and 200.00 µg/mL) achieved by serial dilutions as well as RPMI without any of the named compounds as untreated (control). The treatments were done for 48 h. After 48 h, each plate was subjected to Sulforhodamine B (SRB) assay [59]. Total protein content of viable cells was assessed in this assay. The plate was shaken on an ELISA microplate reader (Tecan, Männedorf, Switzerland) for 5 min and the absorbance was read at 492 nm. The percentage of inhibition was calculated as the following:

(1) $$\mathrm{Percentage}\;\mathrm{of}\;\mathrm{inhibition}\;\left(\%\right)=\frac{{\mathrm{Abs}}_{\mathrm{untreated}}-{\mathrm{Abs}}_{\mathrm{treated}}}{{\mathrm{Abs}}_{\mathrm{untreated}}}\;\times 100\%$$

where Abs_untreated_ = Absorbance of untreated cells at 492 nm; and Abs_treated_ = Absorbance of berberine treated cells at 492 nm.

Graphs of the percentage of inhibition against the concentration were plotted by using GraphPad Prism 7.00 by performing the 4-parameter logistic model (4PL) [60] and the IC_50_ values was determined by using the software. The cells were also cultured in T75 flasks to scale-up the production of HCT116 cells for subsequent analysis and were also used to determine a new IC_50_ value (for berberine).

### 4.5. Cell Count and Growth Curve Analysis

Cell count was done by using Trypan blue (TB) exclusion assay. In total, 100 µL of the cell suspension were pipetted into a microcentrifuge tube and 100 µL of Trypan blue (Nacalai Tesque, Kyoto, Japan) were gently mixed by pipetting in and out. The Trypan blue-dyed cells were then pipetted to fill up both chambers of a hemocytometer (Electron Microscopy Sciences, Hatfield, PA, USA), followed by observation and cell counting under an inverted microscope. Dead cells were stained blue while living cells were not stained. The average number of cells at each time point was used to plot the exponential growth curve following this equation:

(2) $$y=\;y_{0}e^{\lambda t}$$

where *y* = Final number of cells; *y*_0_ = Initial number of cells; *e* = Euler’s number; λ = Growth rate; t = Time that has passed; and the t_d_ was calculated using the following equation:

(3) $$t_{d}=\;\ln2/\lambda$$

### 4.6. Berberine Cellular Localization

Cells were observed by using a Leica DMI6000 B inverted microscope (Leica Microsystems, Wetzlar, Germany) under the phase contrast channel. Without changing the position, the channel was changed to UV to observe berberine fluorescence. To avoid cellular damage by UV radiation, the exposure was kept as minimal as possible, whereby the channel was shut off within 30 s (or less) after capturing the image by using a Digital Color Camera Leica DFC310 FX (Leica Microsystems, Wetzlar, Germany). The corrected total cellular fluorescence (CTCF) was determined by using a free software, ImageJ version 1.52a [61], by using the following equation:

(4) $$\mathrm{CTCF}={\mathrm{IntDen}}_{\mathrm{cell}}-\left(A_{\mathrm{cell}}\times {\bar{X}}_{\mathrm{background}}\right)$$

where IntDen_cell_ = Integrated density of selected cell; A_cell_ = Area of selected cell; and $\bar{X}$_background_ = Average background fluorescence reading.

### 4.7. TELOTAGGG Telomerase PCR ELISA

Protein extraction was done based on the protocol of the *TELOTAGGG* Telomerase PCR ELISA kit (Roche, Mannheim, Germany) with minor adjustments, whereby 5 × 10^5^ cells were used. The protein content was assessed by Bradford Assay (Bio-Rad Laboratories, Hercules, CA, USA) as suggested by the Bio-Rad protocol. The Telomeric Repeat Amplification Protocol (TRAP) reaction was done using a thermal cycler, StepOnePlus™ Real Time-Polymerase Chain Reaction (RT-PCR) Systems (Applied Biosystems, Foster City, CA, USA). In total, 2 µg of lysate were used in this reaction.

### 4.8. RT-PCR

RNA extraction was conducted using the protocol of the ReliaPrep™ RNA Cell Miniprep System (Promega, Madison, WI, USA) with minor adjustments, whereby 1 × 10^6^ cells harvested were lysed and DNase I treatment was done for 1 h 30 min at 30 °C. RNA extracted was quantified by using a NanoDrop™ 2000 (Thermo Fischer Scientific, Waltham, MA, USA). The purity was also assessed using the same instrument based on the A260/280 and A260/230 ratios. RNA integrity was evaluated using 1% native agarose gel electrophoresis containing 5 µL of RedSafe™ (iNtRON Biotechnology, Kirkland, WA, USA) conducted in 1× Tris-Borate-EDTA (TBE) (1st BASE Biochemicals, Singapore Science Park II, Singapore) buffer at 70 V until the tracking dye travelled three-quarters of the gel. Gel picture was visualized, and the image was captured using Gel Documentation System Fusion FX7-7026 (Vilber Lourmat, Marne-la-Vallée, France).

The RNA is said to be intact if the band intensity (28S:18S ratio) is 2:1. Complementary DNA (cDNA) was synthesized using the protocol of the GoTaq^®^ 2-Step RT-qPCR System (Promega, Madison, WI, USA) with minor adjustments, whereby 2500 ng of RNA were transcribed in 20 µL of total reaction mixture. In total, 250 ng of RNA equivalent cDNA were used in RT-PCR reaction mixture and 50 nM of primers (Table 3) were used for each gene. The reaction was conducted using StepOnePlus™ RT-PCR Systems (Applied Biosystems, Foster City, CA, USA) following the cycling conditions shown in Table 4. For no template control (NTC), the cDNA template was replaced with nuclease-free water. For no reverse transcriptase control (NORT), RNA mixture without GoScript™ Reverse Transcriptase, random, and oligo(dT)_15_ primers was used in place of the cDNA template. *CCND1*, *CDK4*, *TERT*, and *TERC* levels were normalized to the geomean of glyceraldehyde-3-phosphate dehydrogenase (*GAPDH*) and *ACTB*.

### 4.9. Relative Telomere Length Quantification

Genomic DNA (gDNA) extraction was conducted using the protocol of the Quick-DNA™ Miniprep Plus Kit (Zymo Research, Irvine, CA, USA). In total, 1 × 10^6^ harvested cells were used for the gDNA extraction. A NanoDrop™ 2000 was used for gDNA quantification and quality assessment. The quantification of the relative telomere length was done according to Vasilishina et al. [62] with minor adjustments, whereby 10 ng of gDNA and a GoTaq^®^ 2-Step RT-qPCR System were used. The amplification was done for 40 cycles and conducted using StepOnePlus™ RT-PCR Systems. Single copy gene (SCG), interferon beta 1 (*IFNB1*) was used for normalization.

### 4.10. Western Blot

In total, 1 × 10^6^ harvested cells were lysed in 60 µL Radio Immunoprecipitation Assay (RIPA) lysis buffer containing 1% protease inhibitor cocktail, sonicated on ice bath for 1 min, incubated for 30 min on ice, followed by centrifugation at 14,000× *g* for 15 min to pellet the cell debris, and subjected to protein estimation by employing Bradford Assay. Then, 20 µg of total protein denatured at 70 °C were separated on 4% and 12% SDS-polyacrylamide stacking and resolving gels, respectively. Electrophoresis was done at 80 V for 30 min, followed by 100 V for 1 h 40 min using pre-chilled (4 °C) 1× SDS running buffer, followed by protein transferring onto 0.22-µm nitrocellulose membrane (Pall Corp., Morelos, Mexico). Electroblotting was conducted using a Mini-Protean^®^ Tetra System (Bio-Rad Laboratories, Hercules, CA, USA) with pre-chilled (-20 °C) 1× SDS transfer buffer and ice pack (−80 °C) for 1 h 40 min on ice. The membrane was removed and incubated in Blocking One (Nacalai Tesque, Kyoto, Japan) for 1 h at room temperature, on a shaker at 100 rpm. The membrane was incubated for at least 13 h in a cold room (5–10 °C) and was constantly shaken at 90 rpm in monoclonal primary antibody at 1:1000 dilution for ACTB and 1:100 dilution for CCND1, CDK4, and TERT, respectively, in Blocking One. The membrane was then washed 3 times with 1× Tris-buffered saline containing 0.1% Tween 20 (TBST), 5 min each time, and on a shaker at 120 rpm. The membrane was incubated on a shaker at 90 rpm with m-IgGκ BP-HRP at 1:10,000 dilution in Blocking One for 2 h at room temperature. The 1× TBST washing step was repeated as previously stated. The membranes were incubated in chemiluminescence solution of Western Bright Sirius (Advansta, San Jose, CA, USA) following the manufacturer’s protocol before the membrane was visualized, and the image was captured using Gel Documentation System Fusion FX7-7026 (Vilber Lourmat, Marne-la-Vallée, France). ACTB was used as the loading control. Protein signal intensities were analyzed by using ImageJ version 1.52a. CCND1, CDK4, and TERT protein signal intensities were normalized to the loading control.

### 4.11. Statistical Analysis

All data obtained in this study were subjected to either *t*-test or one-way ANOVA. Statistical values were expressed as mean ± standard error. *p*-values < 0.05, < 0.01, and < 0.001 were considered as statistically significant, very statistically significant, and highly statistically significant, respectively. Mean values were compared using Duncan’s Multiple Range Test (DMRT). Most statistical analyses were conducted using either Microsoft^®^ Office Excel version 365 or IBM SPSS Statistics version 23.

## 5. Conclusions

In summary, our study demonstrated that berberine decreased the telomerase activity in HCT 116 by simultaneous downregulation of *TERT* and *TERC* levels, which resulted in a decrease of the TERT protein level. Berberine induced G_0_/G_1_ arrest by the upregulation of CCND1 and downregulation of CDK4, whereby the effect of berberine on the cell cycle was time dependent. Telomerase activity of HCT 116 was decreased by berberine treatment and the telomere length was also significantly eroded. Elucidation of berberine’s potential as a telomerase inhibitor in CRC would require further extensive and elaborative efforts in the future.

## Figures and Tables

**Table 1:** Effect of different telomerase inhibitor compound treatments on IC_50_. Values are mean ± standard error of 6 replicates. Mean with different letters differ significantly at *p* < 0.05.

Compound	Boldine	Silymarin	Berberine
IC_50_ (µg/mL)	37.87 ± 3.12 ^b^	>200 ^c^	10.30 ± 0.89 ^a^

**Table 2:** Effect of 10.54 µg/mL berberine treatment on HCT 116 growth at different durations. Berberine emitted green fluorescence under the ultraviolet (UV) channel of an inverted microscope. White arrows indicate nuclei appeared as black, round hollows surrounded by green fluorescence. Black scale bar = 100 µm. Yellow scale bar = 20 µm.

Duration (Hour)	Cell Growth		Berberine Cellular Localization		
	Untreated	Treated	Phase Contrast	UV Channel	Merged
0	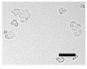	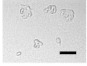	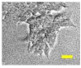	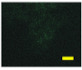	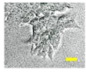
6	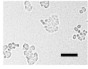	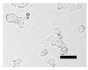	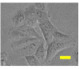	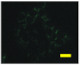	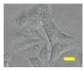
12	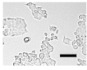	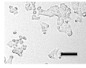	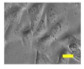	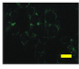	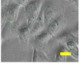
24	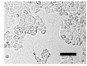	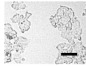	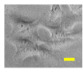	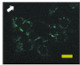	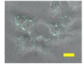
48	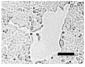	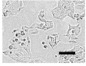	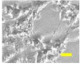	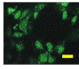	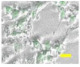
72	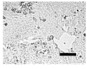	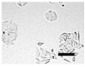	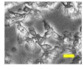	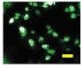	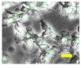

**Table 3:** Primer sequences used in RT-PCR.

NCBI ID	Gene	Forward (5′ to 3′)	Reverse (5′ to 3′)	Amplicon Size (bp)
7015	*TERT*	ACTGCGTGCGTCGGTATGC	CGGCTGGAGGTCTGTCAAGGTA	97
7012	*TERC*	AGAGGAACGGAGCGAGTC	GCATGTGTGAGCCGAGTC	80
595	*CCND1*	AACACGGCTCACGCTTACC	GCCCCATCACGACAGACAAAG	94
1019	*CDK4*	ATGTGGAGTGTTGGCTGTATC	CTGGTCGGCTTCAGAGTTTC	78
2597	*GAPDH*	TTGGTATCGTGGAAGGACTCA	CCAGTAGAGGCAGGGATGAT	133
60	*ACTB*	CGTCTTCCCCTCCATCGT	GCCTCGTCGCCCACATAG	87

**Table 4:** Cycling conditions for RT-PCR.

Step	Cycles	Temperature (°C)	Duration
GoTaq^®^ Hot Start Polymerase activation	1	95	2 min
Denaturation	40	95	15 s
Annealing		53.5	30 s
Extension		60	30 s
Dissociation	1	60 to 95	5 min
Hold		10	∞
